# The financial toll of career-ending concussions in professional hockey

**DOI:** 10.2217/cnc-2016-0008

**Published:** 2016-07-27

**Authors:** Carmen Hiploylee, Richard Wennberg, Charles H Tator

**Affiliations:** 1 Canadian Sports Concussion Project, Krembil Neuroscience Centre, University of Toronto, Toronto Western Hospital, 399 Bathurst Street, Toronto, ON M5T 2S8, Canada

**Keywords:** concussion, cost, hockey, NHL, retirement, salary

## Abstract

**Aim:**

Several high-profile professional hockey players have retired due to career-ending concussions sustained in the National Hockey League (NHL). We sought to determine the salary cost of career-ending concussions in the NHL.

**Methods:**

Concussion-induced retirements from 1995 to 2015 were identified using public media sources. The number of missed games due to a player’s final concussion was multiplied by per game salary to determine cost.

**Results:**

In total, 35 players were identified: the total cost to teams and insurers of career-ending concussions was US$135,476,777. Half of the total consisted of salaries owing three players with contracts still active in 2015.

**Conclusion:**

The financial impact of concussion-induced retirements provides another reason for professional leagues to take measures to better protect their players from concussion.

Concussions suffered in the National Hockey League (NHL) and other professional leagues leading to postconcussion syndrome (PCS) and professional athletes’ premature retirement have been well recognized [1–4], but there has been no report of the financial implications for teams in terms of lost salary costs. To our knowledge, the sole journal report on NHL career-ending concussions consisted of interviews with five NHL players who sustained concussions during their NHL careers [5]. The report revealed the emotional distress, difficulty in transitioning to retirement life and strain on their personal relationships [5]. Although definitions vary, PCS is generally accepted to mean persisting symptoms (somatic, affective or cognitive) due to a concussion that do not resolve within a certain timeframe (10 days to 3 months or more, depending on the definition used) [6,7]. These postconcussive symptoms can be debilitating and can permanently impact the quality of life and health of players [5].

Concussions in professional hockey have received much public and media attention, and it has been stated that a degree of violence deemed intrinsic to the sport is a major contributor to the serious injuries sustained by the players [2,8]. Sociologically, it has been argued that this ‘risk culture’ perpetuates an atmosphere in which pain and injury inflicted on an athlete may be valued and respected over the athlete’s safety and health [9–11]. More recently, this injurious behavior has been increasingly viewed from the perspective of concussions placing athletes at risk of chronic traumatic encephalopathy, a progressive degenerative tauopathy of the brain manifested as dementia and other mental and neurological symptomatology [12].

In 1997, the NHL and the NHL Players’ Association (NHLPA) initiated the NHL-NHLPA Concussion Program and began to collect statistics on concussions in the league. During the seasons 1997–1998 to 2003–2004, 559 physician-diagnosed concussions were tallied during regular season games [13]. Among the 559 reported concussions, 10.6% were diagnosed retrospectively and never evaluated during the game, allowing players to immediately return to play (RTP). Additionally, 8.3% of concussions were evaluated during the game but players were nonetheless cleared for RTP in the same game. For every successive concussion sustained, NHL players took more time to RTP and suffered more symptoms [13].

It is evident that concussions are a problem in the NHL, and this is further exemplified by the number of players who have prematurely ended their playing careers because they were unable to recover from PCS [4]. It is important to explore whether the league and the players have taken sufficient measures to mitigate the problem. One approach to focus attention on the importance of this issue is to investigate the financial implications of career-ending concussions in the NHL. Noncareer-ending concussions can be a significant financial burden as shown by Donaldson *et al.* [14] who found that the total salaries lost from concussions was US$42.8 million a year. Our goal in the present study is novel and involved calculation of the financial loss to the teams and their insurers arising from career-ending concussions in the NHL in the 20-year period from 1995 to 2015.

## Methods

### Identification of concussions

Media reports of concussions sustained in the NHL that led to time lost from play were identified for each player. ‘Game time loss’ has been found to be the most reliable and accurate injury definition to compare injury rates between games, seasons and teams (compared with attempts to identify all injuries, i.e., including those without missed playing time) [15], and the present authors have used this methodology in previous reports [1,4]. Concussions were identified through examination of injury reports published in print or online in *The Hockey News* (Transcontinental Publications, Montreal, QC, Canada) [16], *The Sporting News Hockey Register* (Sporting News Books, Vulcan Sports Media, MO, USA) [17], and *The Sports Network* [18], occasionally supplemented by examination of contemporaneous print or online newspaper or other news media articles, as previously described in detail [1,4]. Since the first paper of Wennberg and Tator [1] other studies have used similar methods to identify NHL injuries [4,14,19–21]. Using these methods, it is important to note that concussions that were either overlooked or did not lead to time lost from play were rarely publicly reported and so the total number of concussions identified for this report is an underestimation [1,4]. Nonetheless, a comparison of the data in the prospective, medical record based analysis of NHL concussions over the seven seasons from 1997 to 2004 by Benson *et al.* [13] with the data obtained from public records in Wennberg and Tator [4] shows nearly identical numbers. Specifically, Benson *et al.* [13] identified 559 total concussions, and ≤534 concussions with missed playing time (30/559 cases were without medical record information on missed playing time [13]), whereas Wennberg and Tator [4] identified 523 concussions with missed playing time over the same time period, a difference in concussion identification of <2%, using the game time loss definition.

### Identification of retired players, inclusion & exclusion criteria

A number of NHL player retirements in the mid-late 1990s, publicly attributed to the effects of PCS, began to attract attention to the career-ending possibilities of PCS, perhaps most notably the premature retirement of Brett Lindros [1,4,22]. In 2007, the late Randy Starkman, a well-respected sports journalist for Canada’s largest daily newspaper, *The Toronto Star*, brought increased attention to this issue with the publication of a list of professional hockey players whose careers were ended by concussion [3]. For this study, we verified, narrowed down (excluding players whose career-ending concussions did not occur in the NHL), and further populated this list through an exhaustive search of all players reported to have had a concussion in the NHL (as defined above), to identify all player retirements and, if reported, the reason(s) for retirement, looking for evidence that a player’s retirement had been publicly attributed to the persistent effects of PCS.

We used the following inclusion and exclusion criteria. To be included, NHL players must have had their career-ending concussion in the NHL. A career-ending concussion was defined as a concussion and ensuing PCS (identified in media reports as persisting debilitating symptoms, most frequently headache and cognitive symptoms) that ultimately caused the player to retire from play in the NHL. Two individuals that had not returned to play for years after a concussion complicated by PCS, but who had not publicly announced their retirement from professional hockey and were still under contract, were considered as retired for the purposes of this study. NHL players meeting our inclusion criteria were identified from 1995 to 2015.

### Retirement & salary cost data collection

All player retirement information and salary data were collected and multiply checked for verification wherever possible from publicly available, hockey-related news sources and websites such as *The Hockey News* [16], *The Sports Network* [18], *NHL.com* [23], *CapGeek* [24], *Blackhawk Zone* [25], *HockeyZonePlus* [26], as well as *Wikipedia* [27] and other print or online newspaper sources.

Player name, total number of reported concussions sustained as an NHL player and information allowing us to calculate the total salary cost due were obtained. The team played for, the player’s age and the year at the time of the last concussion were also recorded.

Total team and league salary payrolls per season were obtained from the salary databases of *HockeyZonePlus* [28] and *USA Today* [29].

### Salary cost due to career-ending concussion

The total salary cost due to a career-ending concussion was calculated for each eligible player in currency worth at the time of the contract signing. This was achieved by multiplying the fraction of missed games over total season games by the total salary for that season, as done previously by Donaldson *et al.* [14]. If the player was not expected to or did not RTP for the remainder of his contract, the remaining salary for the relevant number of contract years was added to the total for the season in which the last game was played.

## Results

### Age & previous concussions

Among the 37 players meeting our inclusion criteria (Table 1), the average age of sustaining a career-ending concussion in the NHL was 31.5 ± 4.5 years and the average number of publicly reported (defined above) concussions sustained as an NHL player was 2.8 ± 1.8.

**Table 1.** **Total salary lost due to career-ending concussions in the National Hockey League.**

**Player name**	**Team^†^**	**Year^‡^**	**Age^§^**	**Total number of concussions^#^**	**Salary cost (US$)**
Brett Lindros	New York Islanders	1995	19	3	4,319,512.20

Dennis Vaske	New York Islanders	1997	30	3	576,219.51

Nick Kypreos	Toronto Maple Leafs	1997	30	1	400,000.00

Pat LaFontaine	New York Rangers	1998	33	3	878,048.78

Jayson More	Nashville Predators	1998	29	1	512,195.12

Warren Luhning	Dallas Stars	1999	24	1	315,548.78

Jeff Beukeboom	New York Rangers	1999	33	2	804,878.05

Geoff Courtnall	St. Louis Blues	1999	37	3	3,243,902.44

Trevor Halverson	Washington Capitals	1999	29	1	400,000.00

Brad Werenka	Calgary Flames	2000	31	1	5,123,170.73

Petr Svoboda	Tampa Bay Lightning	2000	34	5	3,901,897.18

Stu Grimson	Nashville Predators	2001	36	1	1,434,146.34

Cam Stewart	Minnesota Wild	2001	29	2	500,000.00

Adam Deadmarsh	Los Angeles Kings	2002	27	3	4,902,439.02

Mike Richter	New York Rangers	2002	36	2	7,170,731.71

Gino Odjick	Montreal Canadiens	2002	32	3	750,000.00

Ken Belanger	Los Angeles Kings	2002	28	4	878,048.78

Steve Dubinsky	St Louis Blues	2003	36	1	109,756.10

Steve Moore	Colorado	2004	25	1	67,378.05

Eric Lindros	New York Rangers	2004	30	6	1,247,560.98

Scott Stevens	New Jersey Devils	2004	39	2	3,627,074.65

Keith Primeau	Philadelphia Flyers	2005	34	5	9,124,634.15

Rob Dimaio	Tampa Bay Lightning	2006	39	3	500,000.00

Dave Scatchard	Phoenix Coyotes	2007	30	2	1,779,878.05

David Tanabe	Carolina Hurricanes	2007	27	2	1,243,902.44

Matthew Barnaby	Dallas Stars	2007	33	1	274,390.24

Paul Kariya	St Louis Blues	2009	35	3	439,024.39

Kurt Sauer	Phoenix Coyotes	2009	28	2	5,228,658.54

Ian Laperriere	Philadelphia Flyers	2010	36	6	2,200,000.00

Kim Johnsson	Chicago Blackhawks	2010	33	3	904,878.05

Andy Mcdonald	St Louis Blues	2011	34	8	2,612,195.12

Michael Sauer	New York Rangers	2011	24	1	2,134,146.34

Marc Savard^¶^	Boston Bruins	2011	33	6	24,052,439.02

Chris Pronger^¶^	Philadelphia Flyers	2011	37	2	25,189,024.39

Ryane Clowe^¶^	New Jersey Devils	2014	31	5	18,631,098.00

**Total: US$135,476,777**					

^†^Team the player played for when career-ending concussion occurred.

^‡^Year the career-ending concussion was sustained by player.

^§^Age of player when career-ending concussion was sustained.

^#^Total number of reported concussions during the player’s NHL career that led to time lost from play.

^¶^Player contract still in effect in 2015.

### Total salary cost

A total of 37 players met our inclusion criteria. Salary information could not be found for two of these players – Jim Johnson and Jeff Kealty – who had their last concussion in 1997 and 1999, respectively. The financial loss based on salary costs for each of the remaining 35 players is listed in Table 1. The total financial cost for the 35 players’ career-ending concussions from 1995 to 2015 was US$135,476,777. Of note, the salary costs of just three players – Marc Savard, Chris Pronger and Ryane Clowe – contributed 50% of this total loss.

## Discussion

The aim of this study was to determine the financial impact of missed games or seasons due to career-ending concussions in the NHL. For a number of reasons, it is highly likely that the total loss of US$135,476,777 does not reflect the true financial cost of career-ending concussions. For example, this sum does not include the cost of any legal settlements (e.g., Steve Moore received an unknown sum in a lawsuit settlement related to his career-ending concussion [30]), nor does it include the costs of disability insurance or the costs of injury treatment and management.

One might speculate that some of the players included in our study, due to their concussion histories, may have been perceived as ‘less valuable’ or ‘higher risk’ by the time of their career-ending concussion, although this cannot be proven. It might explain why many of the identified concussion-induced NHL retirees did not have high-paying, long-term contracts at the time of their retirement, as some of these players may have been thought unlikely to have enough productive years remaining in their careers to justify a higher salary or longer term contract. By contrast, the salaries of just three of the players on our list – Marc Savard, Chris Pronger and Ryane Clowe – contributed 50% of the overall cost calculated in this study. These players were considered to be in their prime and signed to high-paying and long-term contracts before they suffered their career-ending concussions early in their NHL contract periods.

An unexpected finding in our study was the apparent decline in career-ending concussions after 2011 – the same year Savard and Pronger sustained their career-ending concussions. One possible explanation is that rule changes to make intentional hits to the head illegal in 2010–2011 and 2011–2012 (originally introduced as Rule 48) and the downward trend to employ enforcers [31] may have mitigated the incidence of concussion and concussion-induced retirement. However, Donaldson *et al.* [32] argued that these changes were not effective in reducing concussions. Another possible explanation is that the major financial loss suffered from Savard’s and Pronger’s career-ending concussions in 2011 could have led to a reluctance on the part of some teams – perhaps especially teams with limited salary cap space – to offer long-term contracts to players perceived to be vulnerable based on a history of multiple concussions [33]. Players in this position may have been relegated to other professional leagues where they would eventually retire. Finally, the 2012–2013 NHL lockout and its shortened season may also have contributed to the drop in career-ending concussions since 2011.

Ticket prices along with other arena activities supply 75% of the NHL’s revenue [34]. Sociologically, it has been argued that the NHL’s promotion of violence may be strategic because it has been found that teams that fight more attract larger fan bases, and thus, the violent culture perpetuated by the NHL may allow its teams to profit at the expense of the health of their employees [35]. It is important to note, however, that only approximately 8% of concussions in the NHL are due to fighting [36].

We were not able to apportion the actual financial loss of US$135,476,777. The proportion lost by the league itself, the NHL team owners and their insurance companies, is affected by a variety of factors. There is no publicly available knowledge of the cost of insuring each NHL player and whether or not the individual players identified were insured. It is known that the league requires its teams to insure their top five salary players but the decision to insure the remaining contracts of rostered players is left to the individual teams [37]. We also do not know whether each career-ending concussion listed in our study qualified for insurance, though it has been reported that the injury must result in 30 missed games for insurance coverage to be activated [38], in which case insurance companies are required to pay 80% of the salary [37]. It is not known if, or to what extent, insurance companies may restrict coverage to players based on their concussion histories, or how this may dictate the salary and length of future NHL contracts offered [37].

### Limitations

There are significant limitations to our study. First, the list of NHL players with career-ending concussions in Table 1 is almost certainly incomplete. For example, former NHL player Dean Chynoweth is not included on our list because he had no publicly documented concussions with lost time from play during his NHL career. However, postretirement newspaper reports state that he allegedly sustained 13 concussions in the NHL, and that PCS was the cause of his retirement [3]. Another former NHL player, Jeff Brown, has been reported to have retired from professional hockey because of PCS [39], but no public injury information could be found to verify this. Whether his concussion had resulted in missed games (unlikely given the absence of documentation in any of our sources) or whether it was even sustained as an NHL player remains unknown. Thus, Jeff Brown was not included on our list, and it is likely that we overlooked many other players like him. In other cases, the link between concussion and retirement was not clear for some players and they were therefore excluded from our conservative financial analysis. Many less well known players may not have publicly announced their retirement as being related to concussion and would not have been detected by our methods.

It should be mentioned that many retired former NHL players involved in a lawsuit initiated in 2013 are only now revealing concussion-related symptoms. It is estimated that more than 200 former players are involved and as this lawsuit unfolds, the number of NHL alumni who claim they suffered a career-ending concussion may grow [40]. Incomplete historical concussion information has been attributed to difficulties that players may have in accurately recalling their concussion histories, and also to some players’ desire to hide concussive symptoms during their careers in order to continue playing [41,42].

Another potential limitation is that we acquired all of our data on injuries from publicly available sources, although these have been found to be quite accurate and reliable, a finding that has been attributed to the premise that the injuries suffered by professional athletes are a principal component of the overall entertainment package of professional sports [1,43]. The ability of an NHL team to hide from the public that a player has missed games due to a concussion in this era of sports media saturation must be considered extremely limited [4]. Publicly available salary information, however, may have been less complete, for example, many contracts might have included incentive clauses for performance that were not captured by our methods. Exclusive reliance on public resources also limited the scope of data we could collect: information about the mechanism of injury, the ‘perpetrator’ player or team was often missing. In the data that were obtainable, we did not see a general pattern where a specific team or player was inordinately responsible for a large number of career-ending concussions in the NHL.

NHL player salaries, on average (and notwithstanding salary caps), have been increasing over time and the final dollar amount calculated in this study did not take this into account.

## Conclusion

Many recommendations have been made by scientists and clinicians to reduce concussion incidence and improve concussion management in the NHL. These include increasing the rink size to international standards [44–46], increasing accountability and fines, zero tolerance for deliberate head hits and having independent physicians diagnose concussions rather than team doctors to avoid conflicts of interest [42]. The NHL bears some responsibility for employee safety and well-being in the workplace, and the present paper shows that there is a major financial loss associated with career-ending concussions. The total NHL player salary payroll over the study period amounted to more than US$24.2 billion, and the league’s 2014–2015 player salary payroll alone was greater than US$1.8 billion. Against these numbers, a loss of US$135 million may seem financially acceptable to the teams, league, insurers and even players – part of the ‘cost of doing business’. Nonetheless, the impact of career-ending concussions on players is of course not only financial: retirees with PCS can have significant physical and mental health issues including chronic headaches, anxiety and depression and difficulty transitioning to posthockey life [5]. Moreover, there are continued concerns about the long-term consequences of concussions, specifically, the development of chronic traumatic encephalopathy and related neurodegenerative disorders that have been identified in former professional athletes [12]. The financial impact of concussion-induced retirements is one more reason for professional leagues in the future to take measures to better protect their players from concussion.

**Executive summary**

- Several high-profile professional hockey players have retired due to career-ending concussions sustained in the National Hockey League (NHL) or other professional leagues.
- The emotional turmoil and impact on the careers and lives of these players is substantial but the financial implications to teams, leagues and their insurers are unknown.
- We sought to determine the salary cost of career-ending concussions in the NHL.

**Methods**

- Concussion-induced retirements from 1995 to 2015 were identified using public sports-related media sources.
- The number of missed games due to a player’s final concussion was multiplied by the player’s per game salary to determine the salary cost of each career-ending concussion.
- The age, year and team the player was on when the career-ending concussion occurred were recorded along with the total number of concussions sustained in the NHL.

**Results**

- A total of 37 players were identified to have had career-ending concussions in the NHL from 1995 to 2015.
- Two of these players could not be included in the financial analysis because public salary information was not available.
- The total salary cost to teams and insurers of career-ending concussions was US$135,476,777.
- 50% of this total consisted of the salaries owing three players whose contracts were still active in 2015.
- The average age at career-ending concussion was 31.5 ± 4.5 years and the average number of concussions as an NHL player was 2.8 ± 1.8.

**Discussion**

- More than US$135 million in salary was lost due to career-ending concussions during the study period.
- The parties primarily impacted financially presumably represent a combination of teams and insurers.
- Three players in the early stages of long-term high-paying contracts contributed a large proportion of the final cost.
- There was an apparent decline in concussion-induced retirements after 2011, possibly related to the 2012–2013 NHL lockout and shortened season, increased disciplinary action, decreased fighting or potentially to a growing reluctance of teams to offer contracts to players perceived to be vulnerable to concussion.
- The financial impact of concussion-induced retirements provides another reason for professional leagues to take measures to better protect their players from concussion.
